# Iroquois Family Genes in Gastric Carcinogenesis: A Comprehensive Review

**DOI:** 10.3390/genes14030621

**Published:** 2023-03-01

**Authors:** Everton Cruz dos Santos, Igor Petrone, Renata Binato, Eliana Abdelhay

**Affiliations:** 1 Stem Cell Laboratory, Center for Bone Marrow Transplants, Brazilian National Cancer Institute—INCA, Rio de Janeiro 20230-240, Brazil; 2 Stricto Sensu Graduate Program in Oncology, INCA, Rio de Janeiro 20230-240, Brazil

**Keywords:** gastric cancer, Iroquois, Homeobox

## Abstract

Gastric cancer (GC) is the fifth leading cause of cancer-associated death worldwide, accounting for 768,793 related deaths and 1,089,103 new cases in 2020. Despite diagnostic advances, GC is often detected in late stages. Through a systematic literature search, this study focuses on the associations between the Iroquois gene family and GC. Accumulating evidence indicates that Iroquois genes are involved in the regulation of various physiological and pathological processes, including cancer. To date, information about Iroquois genes in GC is very limited. In recent years, the expression and function of Iroquois genes examined in different models have suggested that they play important roles in cell and cancer biology, since they were identified to be related to important signaling pathways, such as wingless, hedgehog, mitogen-activated proteins, fibroblast growth factor, TGFβ, and the PI3K/Akt and NF-kB pathways. In cancer, depending on the tumor, Iroquois genes can act as oncogenes or tumor suppressor genes. However, in GC, they seem to mostly act as tumor suppressor genes and can be regulated by several mechanisms, including methylation, microRNAs and important GC-related pathogens. In this review, we provide an up-to-date review of the current knowledge regarding Iroquois family genes in GC.

## 1. Introduction

Gastric cancer (GC) is the fifth leading cause of cancer-associated death worldwide, accounting for 768,793 related deaths and 1,089,103 new cases in 2020 [1]. Despite diagnostic advances, GC is often detected in late stages, mainly due to the lack of specificity of symptoms in early stages. The overall 5-year survival is only 20%, and chemotherapy and surgery have limited value in the treatment of advanced cases. In addition, there is a lack of targeted therapies that use molecular markers to treat patients [2,3,4,5]. According to Lauren’s classification [6], gastric adenocarcinoma is divided into two main subtypes: intestinal and diffuse, which differ in their clinical and epidemiological features.

The majority of gastric cancer cases is associated with infectious agents, including *Helicobacter pylori* (present in 65% to 80% of cases) and *Epstein–Barr virus* (EBV) (present in 6% to 10% of cases) [2,3]. While the role of *H. pylori* in the emergence of gastric cancer has already been postulated, the role of EBV is not yet fully understood, and only a small group of patients present with this infection, which has unique molecular alterations and clinicopathological features [4,5,7,8,9].

Many innovative technologies have been used to identify alterations in gastric cancer cell biology. Several genetic abnormalities, such as aberrant gene and protein expression, copy number variation and noncoding RNAs, were identified as possible biomarkers in these studies [10,11,12,13,14]. Moreover, studies to date have indicated that the molecular mechanisms leading to gastric cancer and those responsible for its progression are intricate and not fully understood. Furthermore, the complex relationships among those alterations and insufficient investigation of new molecular findings are challenges to comprehension of the disease that must be overcome.

Iroquois genes are a relatively understudied gene family that encodes a family of transcription factors composed of homeoproteins that are involved in many developmental processes in metazoans [15,16,17]. In mice and humans, there are six Iroquois genes (*IRX1*, *IRX2*, *IRX3*, *IRX4*, *IRX5* and *IRX6*) that have been shown to have important roles during development, for example, in neurogenesis and heart development. Moreover, important developmental pathways that are also related to cancer are associated with Iroquois genes in vertebrates and invertebrates [15,16,18,19,20,21,22]. The study of Iroquois genes and their relationship with cancer is still in the early stages, and few tumors have been used as models for the analysis of these genes. Overall, little is known about the expression and function of the Iroquois gene family in GC. However, given its relationship with important biological pathways, this genic family has unexplored potential to improve our understanding of GC. Thus, in this review, we provide important up-to-date knowledge regarding Iroquois family genes in GC.

## 2. Overview of the Iroquois Gene Family

Homeobox genes are defined by a distinct DNA sequence of 180 bp, the Homeobox, which is present in all metazoans and has been evolutionarily conserved in multicellular organisms. Homeobox genes encode a variable group of proteins possessing a DNA-binding domain of 60 amino acids (exceptions are known) called the homeodomain [23,24,25]. Homeoproteins are mainly transcription factors that activate or repress the expression of target genes, have important roles in cell differentiation and embryonic patterning and are related to human diseases and congenital abnormalities [26].

The Iroquois genes (*Iro*/*Irx*) encode a family of transcription factors composed of homeoproteins that are involved in many developmental processes in metazoans [15,16,17]. *Irx* proteins belong to the TALE superclass of proteins harboring homeodomains that are characterized by the presence, in addition to the homeodomain, of two conserved domains of unknown function called “IRO A” and “IRO box” comprising 13 amino acid residues in the carboxyl-terminal region [15,16,17,27,28]. The *Iro* genes were first discovered in *Drosophila melanogaster*, in which mutations cause bristle formation to be lost on the lateral notum, leaving only a band of bristles in the median part of the notum. This alteration resembles the “Mo-hawk”, common to the Native American tribe “Iroquois”, from which the locus name is derived [17]. In *D. melanogaster*, three genes are grouped in 130 kb of genomic DNA—*araucan*, *caupolican* and *mirror*. Together, they form the Iroquois complex (Iro-C), which is involved in larval development and metamorphosis, the formation of sense organs (including eyes), the specification of the dorsal segment of the adult thorax, the standardization of wing veins, and body segmentation during embryonic development [18,29]. Only one gene, *Irx*, was identified in the nematode *Caenorhabditis elegans* [15], but its function was not characterized. *Irx* genes have also been identified in other species, such as sponges, but were not investigated at the functional level [27,30,31,32].

**Figure 1:**
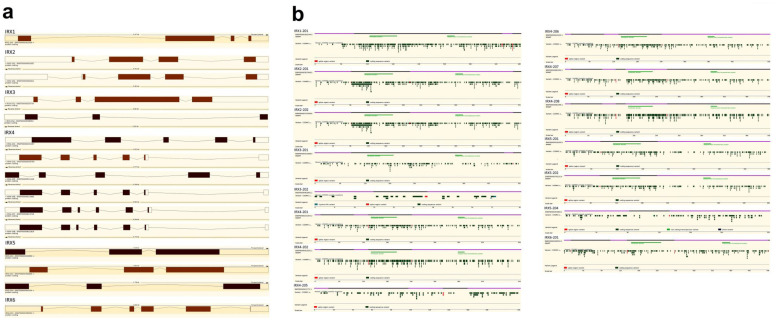
(**a**) Exon/intron structure of 15 transcripts encoding proteins of the Iroquois family. The figure shows representations of the transcript structures. The protein-coding exons are shown as colored rectangles, and introns are shown as lines. The figure information was retrieved from Ensembl (https://www.ensembl.org/index.html, Last accessed on 19 January 2023). A dark brown transcript comes from either the Ensembl automatic annotation pipeline or manual curation by the VEGA/Havana project. (https://vega.archive.ensembl.org/index.html, Last accessed on 19 January 2023). A light brown transcript is identical between Ensembl automated annotation and VEGA/Havana manual curation. (**b**) Protein structure of 15 Iroquois transcripts. Exons are colored as light and dark purple. Light green bars represent the domain location in the protein. The figure information was also retrieved from Ensembl.

Orthologous Iroquois genes have been found in other vertebrates, such as zebrafish, where 11 *Irx* genes are organized in four clusters [22]. In mice and humans, there are six Iroquois genes that are grouped into two genomic clusters (2,2 and 1 Mb) of three genes each: IrxA (on human chromosome 5), which contains the *Irx1*, *Irx2* and *Irx4* genes, and the IrxB cluster (on human chromosome 16), which contains the *Irx3*, *Irx5* and *Irx6* genes [16,33,34]. Iroquois genes have important roles during development; for example, in neurogenesis in *Xenopus laevis*, three Iro genes (*Xiro1*, *Xiro2* and *Xiro3*) were found to participate in the regulation of pro-neural genes in vertebrates during early neurulation [19,35]. Additionally, Iroquois genes are related to development of the kidney [36,37,38], lung [39], pancreatic cells [40], retinal cells [41], female gonads during sex determination [42] and the heart, where all six *Irx* genes (*Irx1*-*Irx6*) are expressed in distinct and overlapping patterns [43,44]. To date, data from the Ensembl human genome database project [45] include one coding protein transcript for *IRX1* (ENST00000302006.4), two for *IRX2* (ENST00000302057.6 and ENST00000382611.10), two for *IRX3* (ENST00000329734.4 and ENST00000558054.1), six for *IRX4* (ENST00000231357.7, ENST00000505790.5, ENST00000511126.1, ENST00000513692.5, ENST00000613726.4, and ENST00000622814.4), three for *IRX5* (ENST00000320990.9, ENST00000394636.9 and ENST00000560154.5) and one for *IRX6* (ENST00000290552.8) (Figure 1a). Data from Simple Modular Architecture Research Tool (SMART https://smart.embl.de, Last accessed on 19 January 2023) retrieved in Ensembl indicates that almost all the transcript harbors both the Homeobox domain (a sequence of 65 amino acids, with exception of *IRX1*, in which the size is 64 amino acids) and the Iroquois-class homeodomain protein (a sequence of 17 amino acids) (Figure 1b). Data from the Catalogue Of Somatic Mutations In Cancer (COSMIC, https://cancer.sanger.ac.uk/cosmic, Last accessed on 19 January 2023), also retrieved in Ensembl, showed that there is no particular hotspot mutation sites through all the somatic variants (Figure 1b).

## 3. Important Pathways Related to Iroquois Genes

Biological alterations observed in cancer are often related to alterations in molecular pathways during signaling cascades. Different pathways are also intimately entangled to form complex regulatory circuits involving several players [46] that are responsible for the fine regulation of multiple metabolic processes. Given the nature of their function as transcriptional factors, Iroquois gene family members may be important players since signal transduction pathways regulate gene expression. In animal models, important highly evolutionarily conserved signaling pathways that govern cellular fate and whose importance for the development of cancer is already well established are associated with Iroquois genes, as we will show below.

In *Drosophila melanogaster*, the *Iro* genes are activated early in the dorsal domain of the ocular disc through Wingless and Hedgehog signaling. It was also discovered that the JAK/STAT signaling pathway positively regulates mirror and possibly also regulates the expression of *araucan*/*caupolican*. Moreover, the Ras/MAPK pathway modulates the transcriptional activity of *araucan*/*caupolican* [47]. *Iro* genes can control cell cycle progression in Drosophila because Iro proteins can downregulate the activity of the Cyclin E/Cdk2 complex [48], which is often lost in tumor cells [49]. In the prospective notum in Drosophila, the early activation of Iro-C seems to be promoted through EGFR. The activator ligand of EGRF, Vein (Vn), is mainly restricted to the prospective notum and might be responsible for Iro-C in this region. Furthermore, the Wnt and BMP-4 signaling pathways are related to the early activation of *Iro* genes in vertebrates [16,18,20]. In the chick, the midbrain develops into the cerebellum through signaling by the FGF8 and mitogen-activated protein (MAP) kinase cascade, which modulates the activity of Irx2 via phosphorylation [50]. In a mouse model to study neural differentiation progress, fibroblast growth factor (FGF) signaling was found to regulate chromatin compaction and nuclear position, where blocking FGFR signaling led to decompaction and a more central nuclear position of the *Irx3* locus [51]. In mice, decreased expression of *Irx2*, *Irx3*, and *Irx5*, markers of the gastric fundus, was observed after disruption of Wnt/β-catenin signaling and resulted in the loss of fundic identity [52], indicating that these genes are also important in stomach development. During the patterning of the proximal and anterior limb skeleton in mice, IRX3 and IRX5 are necessary to initiate the limb bud to specify progenitors of the femur, tibia, and digit and are negatively regulated by Shh signaling [53]. Furthermore, during chick hindlimb development, IRX1 and IRX2 are coordinately expressed and regulated by TGFβ and FGF signaling [54].

**Figure 2:**
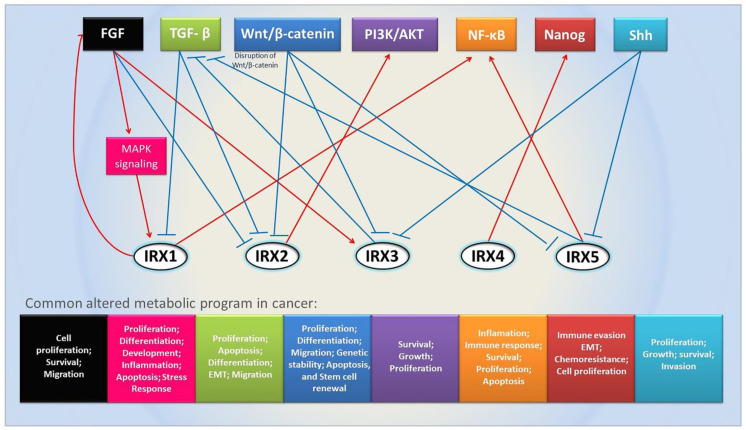
Important signaling pathways related to Iroquois family genes. Arrows indicate a relationship between pathways and Iroquois genes. Blue arrows indicate inhibition, and red arrows indicate activation.

Less information is known about important pathways involving Iroquois genes in cancer. In osteosarcoma (OS), *IRX2* modulates the expression levels of *MMP2* and *VEGF* in an AKT-dependent manner, and overexpression of *IRX2* promotes the activation of PI3K/Akt and increases the proliferation and invasiveness of OS cell lines [55]. The expression of *IRX3* and *IRX5* can downregulate TGF-β signaling in human colorectal cancer cells [56]. In tongue squamous cell carcinoma, increased expression of *IRX5* is a pro-malignant feature that exerts its role through induction of the NF-kB pathway [57]. In non-small cell lung cancer, increased expression of *IRX4* was found to be related to gefitinib resistance and induction of NANOG-mediated responses [58]. Overexpression of *IRX1* in osteosarcoma cells (ZOS and MNNG-HOS) increased the degradation of IκBα and the nuclear translocation of NF-κB p65 [59]. A summary of important pathways related to the Iroquois family discussed above is presented in Figure 2.

## 4. Iroquois Family Genes in Cancer

Iroquois gene studies in cancer are still in their initial phase; thus, few tumors have been used as models for molecular analysis of this genic family, focusing mainly on *IRX1*. In head and neck squamous cell carcinoma (HNSCC), the promoter region of *IRX1* was found to be mutated in 45% of analyzed patients, and its decreased expression was identified. After the overexpression of *IRX1* in HNSCC cells, a decrease in colonies and a reduction in cell number were observed, indicating the tumor suppressor activity of this gene [60]. Aberrant methylation of *IRX1* was also found in lung cancer and correlated with smoking and p53 mutation. Furthermore, patients with *IRX1* methylation showed longer survival and its overexpression in cell lines significantly inhibited cell growth and migration [61,62].

In contrast to previous models, in cervical cancer, high expression of *IRX1* was found to be correlated with tumor staging [63]. In glioblastoma, similar results were found, in addition to being positively correlated with global survival in patients [64]. Increased expression of *IRX1* was also observed in osteosarcoma cell lines and tissues and was strongly related to promoter hypomethylation. Furthermore, negative modulation of *IRX1* in osteosarcoma cell lines profoundly decreased metastatic activity, including migration, invasion and resistance to anoikis, in vitro, and in murine models, it was related to lung metastasis. This pro-metastatic effect of increased *IRX1* expression seems to be mediated by CXCL14/NF-κB signaling activation [59].

In osteosarcoma, increased expression of *IRX2* was observed, indicating that it acts as an oncogene that induces proliferation and invasion [65]. Furthermore, *IRX2* promotes the expression of *MMP*-9 and *VEGF* through PI3K/Akt signaling activation in osteosarcoma cell lines [55]. However, in breast cancer (BC), low expression of *IRX2* was correlated with tumor staging, lymph node status, negative hormonal receptors status and basal-type primary tumors. Overexpression of *IRX2* in BC basal cell lines suppressed the secretion of pro-metastatic chemokines and inhibited cellular mobility [66].

In prostate cancer (PC), quantitative trait locus (QTL) mapping using genome-wide association studies (GWAS) identified risk SNPs for PC in *IRX4* [67]. When *IRX4* was silenced in PC cell lines, cell growth was observed. The opposite effect was observed when overexpression of *IRX4* was induced, indicating tumor suppressor activity of *IRX4* in PC [68]. In pancreatic cancer, decreased expression of *IRX4* was observed in 12 cell lines through hypermethylation of its promoter. It was also observed that the promoter region of *IRX4* was frequently and specifically hypermethylated in pancreatic primary tumors. Moreover, the re-expression of *IRX4* in pancreatic cancer cell lines was able to inhibit colony formation and cell proliferation [69].

Increased expression of *IRX3* and *IRX5* was found in the transition of intestinal adenoma to colorectal cancer, negatively regulating the DPp/TGF-ß pathway [56]. In acute myeloid leukemia (AML), increased expression of *IRX3* was also observed. *IRX3* expression alone was sufficient to immortalize stem cells and hematopoietic progenitors in myeloid culture and to induce lymphoid leukemias in vivo [70]. *IRX5* activity is also present in late blastema/early epithelial stromal and proliferative cells in the developing kidney and Wilms tumors. The *IRX3* expression pattern suggests its involvement mainly in the maturation of nephrogenic structures, with a role in promoting differentiation in Wilms tumors [71].

Decreased levels of *IRX6* were observed in colon adenocarcinoma and rectum adenocarcinoma compared to normal tissues, and *IRX6* had significant prognostic value in discriminating between high- and low-risk patients [72]. Moreover, *IRX6* is a methylation-regulated differentially expressed gene in colon cancer [73]. In luminal-B breast cancer tumor tissues compared with adjacent tissues, *IRX6* was found to be the top 10 downregulated mRNA [74]. Comparing trastuzumab-resistant (SKTR) and trastuzumab-sensitive (SK) HER2+ human breast cancer cell models, *IRX6* was found to be a differentially methylated and differentially expressed gene, indicating a potential role in trastuzumab resistance [75]. In pulmonary carcinoid tumors, *IRX6* was in the top 40 significantly mutated genes [76].

## 5. Iroquois Family Genes in Gastric Cancer

Overall, little is known about the Iroquois genes in cancer, and even less is known about them in GC. As we will see below, almost all information available about Iroquois genes in GC, with the exception of *IRX1*, is restricted to gene expression, which demonstrates that this gene family is still somehow unevaluated in the scientific literature.

### 5.1. IRX1

*IRX1* is the most studied Iroquois gene in GC cancer, and it was first associated with this disease by Lu and colleagues [77], who identified a high frequency of allelic loss in the short arm of chromosome 5 (5p15.33), where *IRX1* was identified. In a study by Yu and colleagues [78] evaluating the relationship between loss of heterozygosity (LOH) of 5p and GC histological phenotype, a deletion region at 5p15.33 was found covering three candidate genes, including *IRX1*, that was mainly related to the IGC subtype. The expression of *IRX1* was reduced or lost in some GC tissues and cell lines; however, no mutations were found in the coding region of the gene [79,80]. Nevertheless, single nucleotide polymorphisms (SNPs) in *IRX1* were identified as GC risk-associated SNPs through genome-wide association analysis of 50 GC samples and 50 normal controls [81]. Additionally, hypermethylation of the promoter region in *IRX1* was detected in cell lines and in the primary tissue and peripheral blood of patients [79], and *IRXI* was found to be hypermethylated in more than two TCGA GC subtypes, namely, chromosomal instability (CIN), microsatellite instability (MSI) and EBV [82]. Furthermore, the hypermethylation of *IRX1* was found to be associated with lymph node metastasis status in early gastric cancer [83], highlighting its potential as a biomarker. Moreover, restoring *IRX1* expression can reduce growth and invasion both in vivo and in vitro, calling attention to its function as a tumor suppressor gene [79]. In this regard, a vasculogenic mimicry (VM) model established using the SGC-7901 gastric cancer cell line showed that in human umbilical vein endothelial cells, tubular formation was significantly inhibited in *IRX1*-transfected cells [84]. Additionally, *IRX1* seems to play an important role in metastasis inhibition: nude mice overexpressing *IRX1* showed marked suppression of peritoneal nodules and pulmonary metastasis via anti-angiogenesis and anti-VM mechanisms. Moreover, cell growth, invasion and tubular formation of human umbilical vein endothelial cells (HUVECs) were increased by *IRX1* overexpression [85].

*H. pylori* is an uncontroversial causal agent of GC [86] and, in 1994, was the first bacterium formally recognized as a human carcinogen type 1 in GC by the International Agency for Research on Cancer (IARC) [87]. A relationship between *H. pylori* and *IRX1* was observed by Guo and colleagues [88], who identified that the levels of *IRX1* promoter methylation in tissue DNA from Hp+ chronic gastritis were higher than observed in Hp- chronic gastritis tissues, 55.30% ± 13.17 versus 5.20% ± 6.31, respectively (*p* < 0.01). They also demonstrated that *H. pylori* infection induced promoter methylation and reduced *IRX1* expression once a higher level of methylation at 12 CpG sites in the promoter region of *IRX1* was observed in the GES-1 human gastric epithelial cell line after *H. pylori* infection compared to the *H. pylori* negative control [88]. Infection with *H. pylori* and inactivation of *IRX1* genes via promoter methylation or allelic loss seem to be common events related to *IRX1* in the development of gastric carcinogenesis.

The molecular mechanisms of *IRX1* are complex, and its regulation by other biomolecules is still unclear. Notwithstanding, Zhi and colleagues [89] found that another epigenetic mechanism can be a regulator of *IRX1*: in a study involving several GC cell lines, they identified that oncogenic miRNA-544 directly targets the 3′-UTR of *IRX1* and that its overexpression causes inactivation and low expression of *IRX1* protein. BDKRB2, a downregulated gene associated with angiogenesis in *IRX1*-transfected gastric cancer cells, is a downstream target gene of *IRX1* and was identified as a potential target for anticancer strategies [85]. Similarly, the inhibition of a microRNA upregulated in GC cells, miR-150, which targets *IRX1* sequences, restrains proliferation, migration, and invasion while facilitating apoptosis of gastric cancer cells by upregulating *IRX1* and represses tumor growth in vivo [80]. Furthermore, the arginine methyltransferase 5 (PRMT5) protein was identified as a major upstream regulator of *IRX1* in GC. The expression of *PRMT5* and *IRX1* revealed opposite correlations at the RNA and protein levels in both gastric cancer cell lines and tissues. Furthermore, PRMT5 promotes methylation of the *IRX1* promoter region by recruiting DNMT3A [90].

### 5.2. IRX2

IRX2 is located on chromosome 5 and was identified in the same study that first identified a correlation between *IRX1* and GC through identification of a high frequency of allelic loss in the short arm of chromosome 5 (5p15.33) [77]. Additionally, *IRX2* was found to be a candidate gene implicated in carcinogenesis of intestinal-type gastric carcinoma during evaluation of the relationship between loss of heterozygosity (LOH) of 5p and GC histological phenotype [78].

Regarding pathological aspects, the hypomethylation of *IRX2* was found to be associated with lymph node metastasis status in early GC patients [83].

Decreased expression of *IRX2* was observed in biopsies of a Norwegian GC population [91]. Downregulation of *IRX2* was also observed in *H. pylori*-positive atrophic gastritis compared with normal gastric mucosal tissues, and in the comparison between *Helicobacter pylori*-positive atrophic gastritis and *Helicobacter pylori*-positive GC, it was found to be upregulated. These results show that *H. pylori* infection, one of the most important gastric cancer risk factors, is closely related to *IRX2* expression before and during malignant transformation [92] and indicate that *IRX2* expression is mediated by complex unknown regulatory events.

*IRX2* was identified together with other genes as a GC immune-related transcription factor (IRTF) in a complex analysis of 1136 gastric cancer samples, in which IRTF regulation patterns were associated with clinical phenotypes, tumor immune microenvironment, immunogenicity and prognosis in GC [93]. *IRX2* was found to be an important transcription factor that was downregulated in a regulatory network study of 210 gastric cancer tissues and 118 normal tissue samples [94]. Interestingly, the expression status of *IRX2* was upregulated in early gastric cancer (EGC) compared with low-grade intraepithelial neoplasia (LGIN), one of the last stages of precancerous lesions [95,96,97], in a study of hub genes regulating EGC in a Chinese (Qinghai) population. Additionally, Vastrad and Vastrad [98] found that *IRX2* was downregulated in samples of diffuse GC, indicating that in both the intestinal and diffuse subtypes, the expression of *IRX2* is altered.

### 5.3. IRX3

Downregulated expression of *IRX3* was observed in a Norwegian GC population and in multiple population studies (Romanian, Singapore, Italy, South Korea, and China) [91,94,99,100,101] and was identified in a transcription factor regulatory network of downregulated hub genes (higher degree of interaction with other genes) in diffuse type GC [98]. *IRX3* was also identified as an IRTF [93], and its downregulated expression was found to be a key element involved in a transcription factor regulation network analysis in Epstein–Barr virus-associated gastric cancer (EBVaGC) [102]. The hub transcription factors *IRX3*, *NKX6*-2, *PTGER3* and *SMAD5* were identified (by in silico analysis) to regulate target genes and may participate in EBVaGC pathogenesis by regulating important genes such as *SST* (Somatostatin), an important regulator of motor activity and the secretion of gastrin-stimulated gastric acid in the gastrointestinal tract [103], and *GDF5* (growth differentiation factor 5), a regulator of differentiation and cell growth in both embryonic and adult tissues, whose aberrant expression is related to cancer [104,105,106,107]. Interestingly, in a model of gastric tumorigenesis using mice deficient in *TFF1* expression, which is an important gene in the protection and repair of gastric mucosa, upregulated expression of *IRX3* was identified in the total gastric RNA [108]. Additionally, *IRX3*, together with *IRX2* and *IRX5*, are key elements related to embryonic stomach pattern formation and embryonic fundus development. The expression of these TFs is more than tenfold enriched in the embryonic fundus compared to the antrum. Additionally, the disruption of Wnt/β-catenin signaling resulted in the loss of fundic identity, with dramatically reduced expression of the fundus markers *IRX2*, *IRX3*, and *IRX5* in mice and humans [52].

### 5.4. IRX4

Chen and colleagues [109] observed that the expression of *IRX4* was modulated in gastric tissues in response to ionizing radiation. They found that *IRX4* was downregulated in the mouse stomach after irradiation with 6 or 12 Gy X-rays, and they also observed that radiation resulted in atrophy of the gastric mucosa and abnormal morphology of chief and parietal cells. Using the TCGA database, Sun and colleagues [110] identified that *IRX4* expression was downregulated in GC patients and associated with their survival outcomes.

### 5.5. IRX5

*IRX5* has been found to be downregulated in gastric cancer samples compared to adjacent tissues in several populations [111]. In a study comparing subcutaneous adipose tissue (SAT) and visceral adipose tissue (VAT) in the same cachectic GC patient, *IRX5* was observed to be one of the top 10 upregulated DEGs, and it was also identified as a key gene in a regulatory network [112]. *IRX5* was identified in diffuse type GC in a transcription factor regulatory network of downregulated hub genes [98] and was identified as a GC IRTF [93]. Downregulation of *IRX5* was observed in *H. pylori*-positive atrophic gastritis compared with normal gastric mucosal tissues, indicating that *H. pylori* infection, one of the most important gastric cancer risk factors, is also related to *IRX5* expression regulation [92]. *H. pylori* infection was found to be associated with an increase in *IRX5* protein expression in GES-1 (normal human gastric epithelium) cells after *H. pylori* infection and incubation for 24 h [113]. Furthermore, in the MKN45 cell line (established from a poorly differentiated gastric adenocarcinoma), *IRX5* (*IRX-2a*) expression was found to be upregulated after treatment with *H. pylori* water extract [114]. Compared with the above discussions in the previous sections concerning *IRX1* and *IRX2*, these results indicate that *H. pylori* can differentially modulate different Iroquois family genes.

### 5.6. IRX6

**Figure 3:**
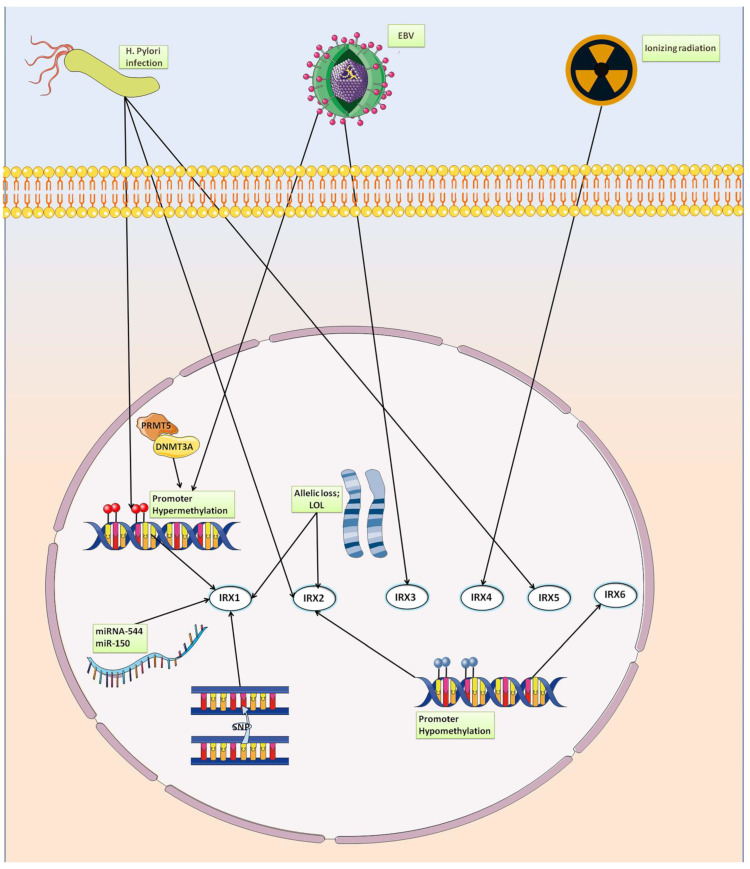
Mechanisms of Iroquois family gene regulation in GC.

The only information regarding this gene in GC disease is the hypomethylation of *IRX6*, which was found to be associated with lymph node metastasis status in early gastric cancer [83]. All information regarding the Iroquois family components in GC discussed above are presented in Table 1. Additionally, a scheme of mechanisms related to the regulation of Iroquois genes in GC is shown in Figure 3.

## 6. Status of Iroquois Family Genes in GC Patient Databases

**Figure 4:**
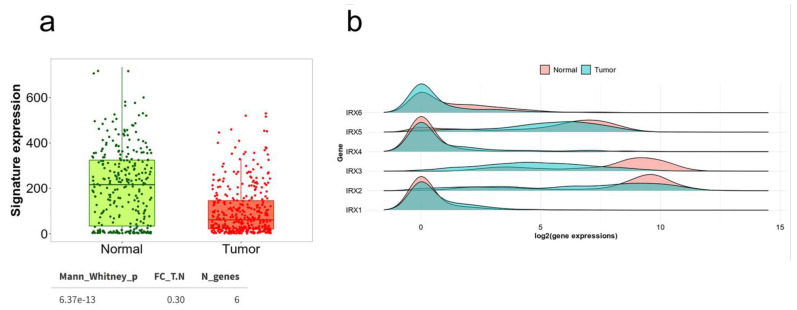
The expression signature of Iroquois genes in GC patients. (**a**) The gene signature analysis page calculates the means of the selected gene signature across each patient one by one and provides a summary plot using RNA-Seq-based data. (**b**) Multiple gene analysis provides an overview of the selected gene set in the selected tissue using RNA-Seq-based data.

Given the lack of information about Iroquois genes in GC, to enrich our scrutiny of this genic family, we thought that investigating data already available in public databases could be very informative and expand our knowledge of these genes. Therefore, we performed a series of analyses using different Web Tools to gather more information about Iroquois genes, mainly in The Cancer Genome Atlas (TCGA) database. The TCGA initiative performed a large study using high-throughput molecular approaches, such as whole-exome sequencing and mRNA sequencing, and was able to identify molecular subtypes of GC [115]. These data are accessible and can be used in different studies. Overall, the expression signature of Iroquois genes in GC patients is downregulated, regardless of subtype, as we can see in the differential gene expression analysis of tumor versus normal tissues performed using the software TNMplot (Figure 4a,b), which uses RNA-Seq data from TCGA for a comparison of gene expression changes among tumor, normal and metastatic tissues [116]. Furthermore, analysis of the expression of Iroquois genes in UALCAN, a database that enables in-depth analysis of TCGA data [117], seems to indicate a relationship with the subtype of GC (Figure S1 (Supplementary Materials)). We also evaluated the differential use of Iroquois isoforms in GC using the GEPIA2 software [118] which utilizes TCGA and Genotype-Tissue Expression (GTEx) data. In this analysis we identified that there is a differential expression of variant isoforms in GC (Supplementary Figure S2), indicating that different isoforms may contribute in different ways to GC carcinogenesis. We also investigate the mutation status of Iroquois genes in GC in the Catalogue Of Somatic Mutations In Cancer (COSMIC) [119]. COSMIC data describes 5,977,977 coding mutations across 1,391,372 samples in which expert scientists have thoroughly analyzed 223 important cancer genes, compiling data from 26,251 papers in the process. Together with open-access data from The Cancer Genome Atlas (TCGA), International Cancer Genome Consortium (ICGC), and merged with genome-wide annotations from 466 whole genome, COSMIC is a reliable source of information. In this database, we verified that, although there were no mutation hotspot sites in Iroquois genes, there is some somatic mutations that were predicted as potentially pathogenic, based on Functional Analysis through Hidden Markov Models algorithm [120]. We also observed that, regarding the mutations present in the domain’s sites, the Homeobox domain showed the majority number of mutations for almost all Iroquois genes (Table 2). Although none of these mutation sites were definitively described as pathogenic in the literature, their presence in the Homeobox domain is an interesting feature that requires further investigation since they may be related to GC carcinogenesis. Finally, we observed that some Iroquois genes with available expression data in the KM plotter database (integrated samples of three major cancer research centers: Berlin, Bethesda and Melbourne datasets and publicly available datasets) [121], are differentially related to OS in gastric cancer regarding subtypes. In combined analysis high expression of *IRX2* and *IRX3* are related to worst OS. In its turn, *IRX4* are related to better OS in IGC and the opposite in DGC (Supplementary Figure S3).

## 7. Conclusions

Given the importance of transcription factor Homeobox genes, such as the Iroquois family, in crucial biological events, including development, and their relationship with several cancer-related pathways, the limited information about their function in cancer, mainly in GC, is an issue that requires attention. This limitation opens a wide range of possibilities, especially with regard to studies based on gene–gene interactions, to identify targets, determine how they are regulated, and investigate how Iroquois genes impact the regulation of biological pathways, since these types of studies are practically nonexistent for the Iroquois family in GC. Thus, scientific effort must be made to explore the potential function of this genic family in GC, which will certainly expand the current molecular knowledge of this disease.

## Figures and Tables

**Table 1:** Iroquois family genes in gastric cancer research.

*IRX1*						
Regulation	Methodology	Metilation Status	Tissue	Histology	Sample	Citation
Down	RT–qPCR	Metilated	GC	Intestinal	NCI-N87 Cell line	[79,80,89,90]
Down	RT–qPCR	Metilated	GC	Poorly differentiated	SNU-1 Cell line	
				carcinoma		
Down	RT–qPCR	Metilated	GC	Diffuse	SNU-16 Cell line	
Down	RT–qPCR	Metilated	GC	Intestinal	AGS Cell line	
Down	RT–qPCR	Metilated	GC	Diffuse	KATO-III Cell line	
Down	RT–qPCR	Metilated	GC	Diffuse	MKN-45 Cell line	
Down	RT–qPCR	Metilated	GC	Intestinal	SGC-7901 Cell line	
Control non cancer	RT–qPCR	Unmetilated	Epitelial	Normal	GES-1 (control cell)	
Control non cancer	Western blot/RT–qPCR		Epitelial	Normal	RGM-1 Cell line	[80]
Down	Western blot/RT–qPCR		GC	Intestinal	BGC823 Cell line	[90]
Down	Western blot/RT–qPCR		GC	Intestinal	MKN28 Cell line	
Down	Western blot/RT–qPCR		GC	Diffuse	Hs746T Cell line	
Down	Western blot/RT–qPCR		GC	Poorly differentiated	SUN-1 Cell line	
				carcinoma		
Down	Western blot/RT–qPCR		CG		36 patients cancer and adjacent	
Down	Western blot/RT–qPCR		GC		60 patients cancer and adjacent	[80]
	genome-wide methylation sequencing	Hypermetilated	GC	GC	23 lymph node metastasis positive (LNM+) and 24 lymph node metastasis negative (LNM−)	[83]
*IRX2*						
Regulation	Methodology	Metilation status	Tissue	Histology	Sample	Citation
Down	cDNA microarrays		GC	Diffuse/intestinal/mixed	20 patients cancer and adjacent	[91]
Up	Gene expression array		EGC, LGIN		58 patients	[95]
Down	Microarray		GC and normal		210 gastric cancer tissues and 118 normal tissue	[94]
Down	LncRNA/mRNA analysis set		GC, normal mucosa and atrophic gastritis		13	[92]
Down	Microarray		GC	Diffuse	107 diffuse type gastric cancer tissues samples and 10 normal gastric tissues samples	[98]
Down	Microarray		GC	GC	210 gastric cancer tissues 118 normal tissue samples	[94]
	Genome-wide methylation sequencing	Hypometilated	GC	GC	23 lymph node metastasis positive (LNM+) and 24 lymph node metastasis negative (LNM−)	[83]
*IRX3*						
Regulation	Methodology	Metilation status	Tissue	Histology	Sample	Citation
Down	cDNA microarrays		GC	Diffuse/intestinal/mixed	20 patients cancer and adjacent	[91]
Down	Microarray		GC	GC	70 gastric adenocarcinoma samples and 68 matched normal samples	[99]
Down	Microarray		GC	GC	663 cancer samples and 223 normal cases	[100]
Down	Microarray		GC	Diffuse	107 diffuse type gastric cancer tissues samples and 10 normal gastric tissues samples	[98]
Down	Microarray		GC	GC	210 gastric cancer tissues 118 normal tissue samples	[94]
*IRX4*						
Regulation	Methodology	Metilation status	Tissue	Histology	Sample	Citation
Down	microarray		Stomach		mice	[109]
Down	RNA-seq		GC	GC	375 GC primary tumor samples and 32 solid normal tissue s	[110]
*IRX5*						
Regulation	Methodology	Metilation status	Tissue	Histology	Sample	Citation
Down	Microarray		GC	GC	63 GC tissues and 56 paracancer tissues	[111]
Down	microarray		SAT and VAT	GC	3	[112]
Down	microarray		GC	Diffuse	107 diffuse type gastric cancer tissues samples and 10 normal gastric tissues samples	[98]
*IRX6*						
Regulation	Methodology	Metilation status	Tissue	Histology	Sample	Citation
	Genome-wide methylation sequencing	Hypometilated	GC	GC	23 lymph node metastasis positive (LNM+) and 24 lymph node metastasis negative (LNM−)	[83]

**Table 2:** Characterization of potentially pathogenic somatic mutations related to Iroquois genes in GC patients.

Transcript Name (Translation ID)	Homeobox Domain			Iroquois-Class Homeodomain Protein			Possible Pathogenic Mutation **	Samples Tested	Mutated Samples	Homeobox Domain Possible Pathogenic Mutation **	Iroquois-Class Homeodomain Protein Possible Pathogenic Mutation **
	Start	End	Exon	Start	End	Exon	Mutation (Amino Acid)				
IRX1-201 (ENST00000302006.4)	128	192	2	309	326	2	p.A71V; p.A114V; p.K132T; p.Y154C; p.A179T; p.A181T; p.A181V; p.R182C; p.R196C; p.I223V; p.E290V; p.S377=; p.R389H; p.V394I; p.S433R; p.V442D; p.D461N; p.N462T; p.G469E; p.R472W	63	19	p.K132T; p.Y154C; p.A179T; p.A181T; p.A181V; p.R182C	-
IRX2-201 (ENST00000302057.6)	114	179	2	322	339	3	p.N113K; p.A116T; p.K130R; p.Y141=; p.T157A; p.R171H; p.D217=; p.E235 *; p.C250G; p.S254L; p.D265A; p.E274 *; p.S386P; p.N399=; p.Y410C; p.E458K; p.V463I	50	16	p.A116T; p.K130R; p.Y141=; p.T157A; p.R171H	-
IRX2-202 (ENST00000382611.10)	114	179	2	322	339	3					
IRX3-201 (ENST00000329734.4)	128	192	2	342	359	2	p.Y73=; p.G158=; p.R184L; p.R184=; p.G203E; p.P336S; p.R463H; p.V481=	33	8	p.G158=; p.R184L; p.R184=	-
IRX4-201 (ENST00000231357.7)	142	207	4	361	378	5	p.A35V; p.T88A; p.P124S; p.S168I; p.T170M; p.R177H; p.I207V; p.R225H; p.T233M; p.A271=; p.R348M; p.S439A; p.P457L; p.S499L; p.A502V	41	14	p.S168I; p.T170M; p.R177H; p.I207V; p.R225H; p.T233M	-
IRX4-202 (ENST00000505790.5)	142	207	4	361	378	5					
IRX4-206 (ENST00000513692.5)	142	207	4	361	378	5					
IRX4-207 (ENST00000613726.4)	168	233	5	387	404	6					
IRX4-208 (ENST00000622814.4)	168	233	5	387	404	6					
IRX5-201 (ENST00000320990.9)	113	178	2	326	343	3	p.P114T; p.T156=; p.L157I; p.R182Q; p.E188G; p.E201K; p.D212N; p.A341V; p.A452T; p.P457=; p.?	25	11	p.P114T; p.T156=; p.L157I	p.A341V
IRX5-202 (ENST00000394636.9)	113	178	2	326	344	3					
IRX6-201 (ENST00000290552.8)	146	211	4	344	361	5	p.V34I; p.A44V; p.R55Q; p.L56=; p.S145C; p.P349L; p.E432K	33	7	-	p.P349L

** The mutation impact filters are derived from the FATHMM-MKL algorithm (Functional Analysis through Hidden Markov Models). FATHMM-MKL is an algorithm which predicts the functional, molecular and phenotypic consequences of protein missense variants using Hidden Markov Models. Where FATHMM-MKL scores are ≥0.7 the mutation is classified as ‘pathogenic’, or ‘neutral’ if the score is ≤0.5; * Mutation type: Substitution—Nonsense; = Mutation type: Substitution—coding silent; ? Mutation type: Unknown.

## Data Availability

The data presented in this study are available in the Supplementary Materials.

## Supplementary Materials

The following supporting information can be downloaded at: https://www.mdpi.com/article/10.3390/genes14030621/s1, Figure S1: Comparison of Iroquois family gene expression in patients with gastric cancer. Gene expression data of Iroquois family genes in patients with GC of different histological subtypes were obtained from TCGA and plotted using UALCAN software (ualcan.path.uab.edu/, accessed on 19 January 2023). To the left of each plot, comparisons between the subtypes that present expression differences with significant values can be observed; Figure S2: Differential expression of IRX2, IRX3, IRX4, IRX5 and IRX6 isoforms in GC versus normal tissue within the STAD TCGA cohort. Bar-plot panel present the isoform usage (from 0% to 100%) distribution (plotted using GEPIA2 software); Figure S3: Overall survival curves related to Iroquois family gene expression levels in GC patients considered as a whole and separated by CGI and CGD subtypes (plotted using KM plotter software).
