# Supplementary material for: Iroquois Family Genes in Gastric Carcinogenesis: A Comprehensive Review
Source: Genes (Basel). 2023 Mar 1;14(3):621. doi: 10.3390/genes14030621 (PMC10048635; doi:10.3390/genes14030621)
Supplement: Supplementary file 1 [file genes-14-00621-s001.zip › genes-2207440-supplementary.pdf]

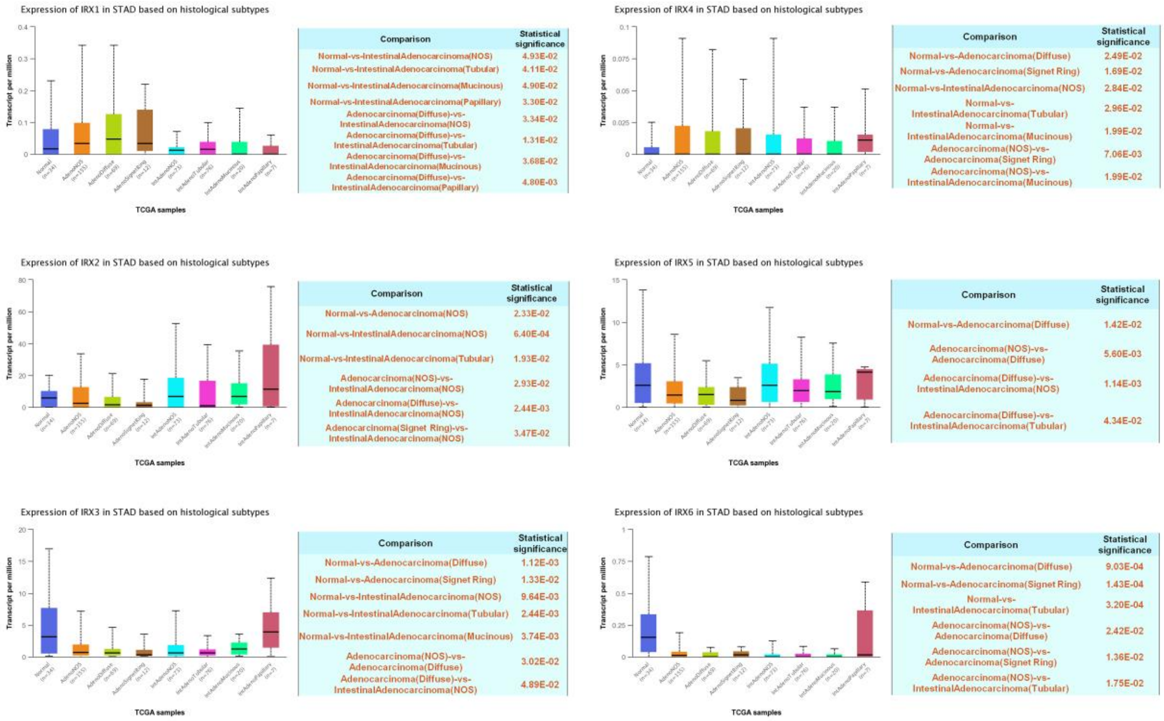

Figure S1. Comparison of Iroquois family gene expression in patients with gastric cancer. Gene expression data of Iroquois family genes in patients with GC of different histological subtypes were obtained from TCGA and plotted using UALCAN software (ualcan.path.uab.edu/). To the left of each plot, comparisons between the subtypes that present expression differences with significant values can be observed.

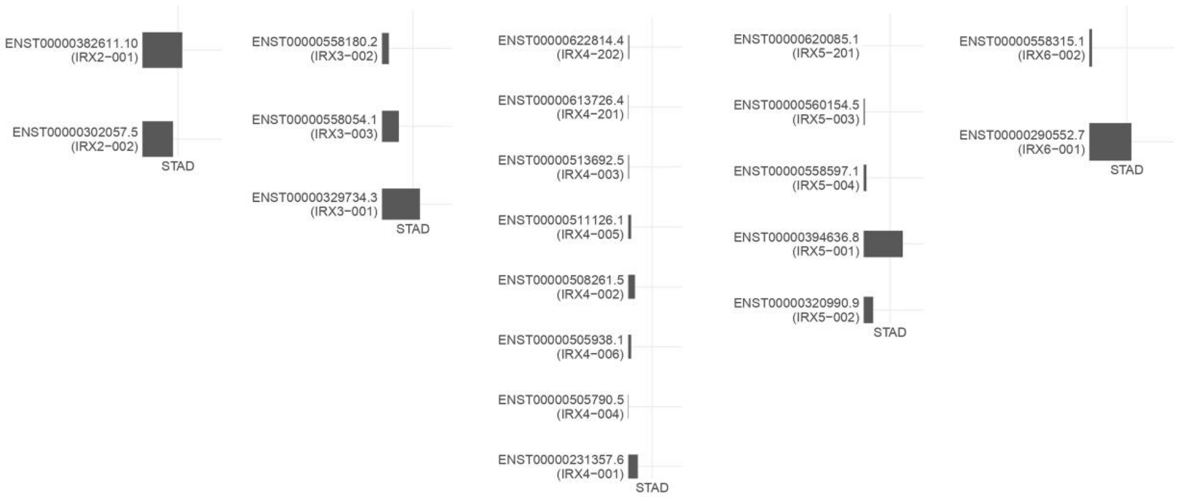

Figure S2. Differential expression of IRX2, IRX3, IRX4, IRX5 and IRX6 isoforms in GC versus normal tissue within the STAD TCGA cohort. Bar-plot panel present the isoform usage (from 0% to 100%) distribution (plotted using GEPIA2 software).

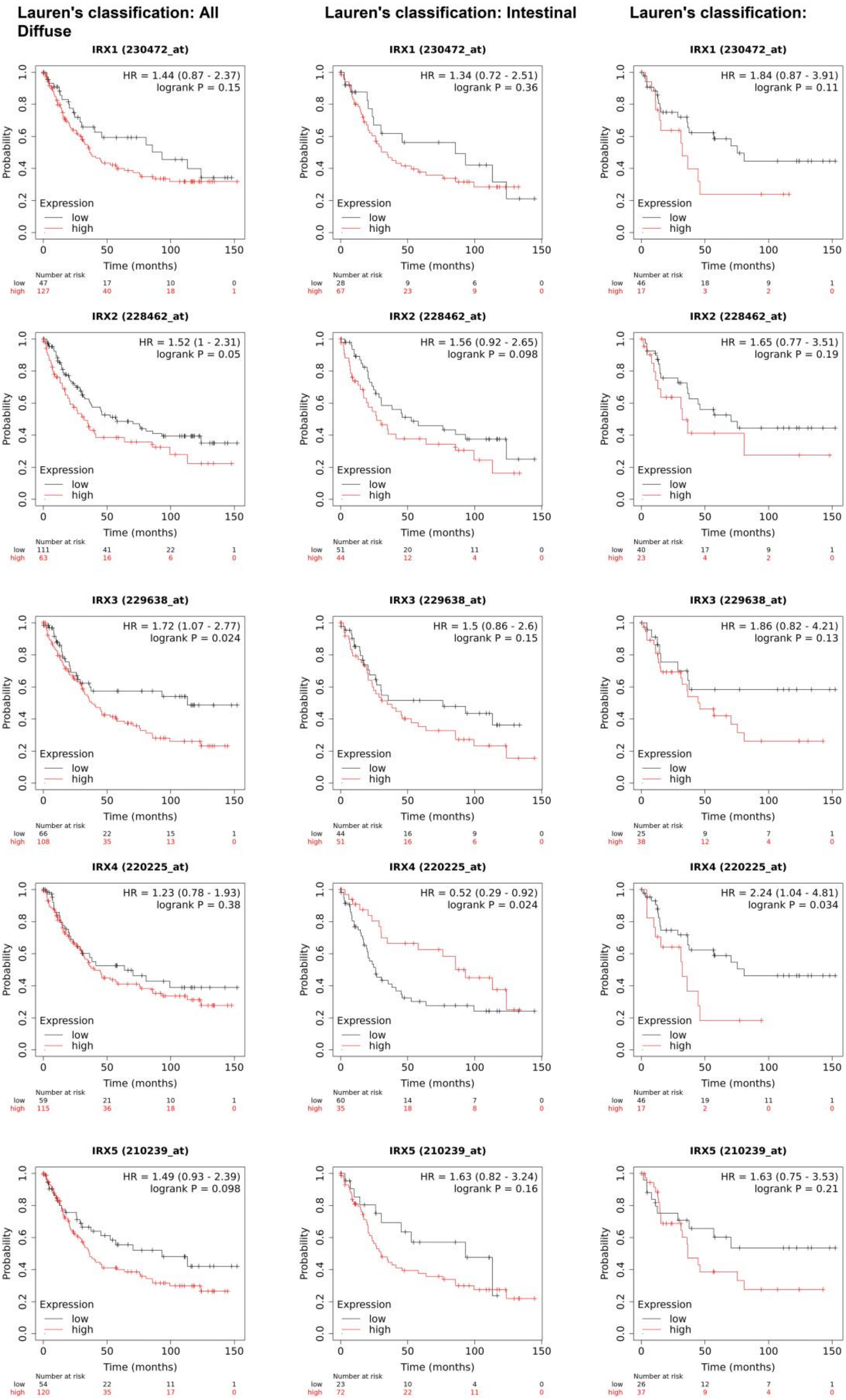

Figure S3. Overall survival curves related to Iroquois family gene expression levels in GC patients considered as a whole and separated by CGI and CGD subtypes (plotted using KM plotter software).
